# P16 Concurrent sacroiliitis and infective endocarditis in a young male with congenital aortic stenosis: a diagnostic challenge and learning points

**DOI:** 10.1093/rap/rkae117.047

**Published:** 2024-11-05

**Authors:** Rania Hassan Elsayed Ali, Clare Warrell, Jessica Manson

**Affiliations:** University College London Hospitals NHS Foundation Trust, London, United Kingdom; University College London Hospitals NHS Foundation Trust, London, United Kingdom; University College London Hospitals NHS Foundation Trust, London, United Kingdom

## Abstract

**Introduction:**

In young patients, severe sacroiliac pain and inflammation often raise suspicion for inflammatory conditions such as ankylosing spondylitis, particularly in the presence of HLA B27 positivity. However, the diagnostic process becomes more complex when these symptoms occur alongside a history of congenital heart disease. This case report discusses a patient with a background of congenital aortic stenosis who presented with acute sacroiliac pain and inflammation that evolved into a diagnosis of infective endocarditis. This case highlights the importance of considering a broad differential diagnosis, including infectious etiologies, in patients with severe back pain and a history of cardiac anomalies.

**Case description:**

A 22-year-old male rugby player with a medical history of congenital mild aortic stenosis, which was treated with balloon dilation at six months, presented with lower back pain radiating to the right gluteal area and back of the thigh for the past 10 days. The pain had a sudden onset and initially resembled a muscle strain, characterised by sharp, shooting pain extending from the lower back to the right foot, exacerbated by movement. Initial management with physiotherapy, NSAIDs, and diazepam provided limited relief. Over the last five days, his condition deteriorated, rendering him unable to move out of bed. Further history revealed no joint pain, swelling, stiffness, or other systemic symptoms commonly associated with inflammatory or infectious conditions. The patient had no significant family of inflammatory disease or social history pertinent to his presenting symptoms. No dental procedures or issues before.

During the physical examination, he had no fever (was on regular paracetamol and ibuprofen). The patient experienced difficulty sitting up from bed and exhibited tenderness in the lower spine. There was no synovitis in peripheral joints, and a straight leg raise test was limited by pain in the lower right back and leg. A cardiac examination revealed a loud S2 and a faint 1+ systolic murmur at the left sternal border—no peripheral sign of infective endocarditis.

Imaging and laboratory investigations revealed active sacroiliitis on MRI, elevated inflammatory markers (CRP 87 & ESR 28), negative autoimmune screen, and the presence of *Streptococcus cristatus* in two different sets of blood cultures. Transthoracic and transoesophageal echocardiograms indicated severe aortic regurgitation with a bicuspid aortic valve, severe left ventricular dilation, and a small echogenic structure suggestive vegetation, pointing towards infective endocarditis. The PET scan showed reactive changes in the spleen and bone marrow. HLA B27 positive, indicating a genetic predisposition to inflammatory conditions.

**Discussion:**

This case underscores the diagnostic complexity of severe sacroiliac pain and inflammation in young patients, particularly when compounded by a history of congenital heart disease and the emergence of infective symptoms. Initially, the patient’s presentation with lower back pain radiating to the gluteal area suggested a musculoskeletal origin, a common initial misinterpretation. However, the lack of response to conventional treatment and the progressive nature of the symptoms necessitated a more thorough investigation, revealing an underlying infectious aetiology.

The MRI findings of active sacroiliitis and identifying *S. cristatus* in blood cultures pointed towards an infectious process involving the sacroiliac joint. Sacroiliitis can present with non-specific symptoms that mimic other inflammatory or mechanical back pain, making diagnosis challenging. In this patient, the presence of severe aortic regurgitation and vegetation on the echocardiogram confirmed the diagnosis of infective endocarditis (IE), a rare but critical consideration in patients with underlying congenital heart defects.

The association between HLA B27 positivity and inflammatory conditions such as ankylosing spondylitis is well-documented. However, the concurrent presentation of sacroiliitis and infective endocarditis in this patient highlights the need for a broad differential diagnosis when evaluating inflammatory back pain, particularly in the context of systemic symptoms or underlying cardiac abnormalities.

*S. cristatus*, although part of the normal oral flora, can be a rare but significant pathogen in IE. The presence of severe aortic regurgitation and left ventricular dilation in this patient underscores the potential for rapid progression and the need for timely intervention.

This case illustrates the importance of considering infective aetiologies in young patients with severe sacroiliac pain, particularly when standard treatments fail and systemic signs of infection are present. Integrating imaging, microbiological, and echocardiographic findings was pivotal in diagnosing and managing this complex case, ultimately guiding appropriate antimicrobial therapy and cardiac care.

**Key learning points:**

• **Consider broad differential diagnoses**: Severe sacroiliac pain and inflammation in young patients, especially those with a history of congenital heart disease, should prompt consideration of both inflammatory and infectious aetiologies.

• I**mportance of comprehensive evaluation**: When initial treatments for presumed musculoskeletal pain are ineffective and symptoms worsen, a thorough evaluation including imaging, laboratory tests, and microbiological cultures is crucial.

• **Role of HLA B27**: The presence of HLA B27 can indicate a predisposition to inflammatory conditions, but it does not exclude the possibility of concurrent infectious processes.

• **Awareness of rare pathogens**: *Streptococcus cristatus*, though uncommon, can be a significant pathogen in infective endocarditis, particularly in patients with predisposing cardiac anomalies.

• **Multidisciplinary approach:** Effective management of complex cases involving multiple systems requires a coordinated approach involving specialties such as cardiology, infectious disease, and rheumatology to optimise patient outcomes.

